# Suicide Risk Assessments Through the Eyes of ChatGPT-3.5 Versus ChatGPT-4: Vignette Study

**DOI:** 10.2196/51232

**Published:** 2023-09-20

**Authors:** Inbar Levkovich, Zohar Elyoseph

**Affiliations:** 1 Oranim Academic College Faculty of Graduate Studies Kiryat Tivon Israel; 2 Department of Psychology and Educational Counseling The Center for Psychobiological Research Max Stern Yezreel Valley College Emek Yezreel Israel; 3 Department of Brain Sciences Faculty of Medicine Imperial College London London United Kingdom

**Keywords:** artificial intelligence, ChatGPT, diagnosis, psychological assessment, psychological, suicide risk, risk assessment, text vignette, NLP, natural language processing, suicide, suicidal, risk, assessment, vignette, vignettes, assessments, mental, self-harm

## Abstract

**Background:**

ChatGPT, a linguistic artificial intelligence (AI) model engineered by OpenAI, offers prospective contributions to mental health professionals. Although having significant theoretical implications, ChatGPT’s practical capabilities, particularly regarding suicide prevention, have not yet been substantiated.

**Objective:**

The study’s aim was to evaluate ChatGPT’s ability to assess suicide risk, taking into consideration 2 discernable factors—perceived burdensomeness and thwarted belongingness—over a 2-month period. In addition, we evaluated whether ChatGPT-4 more accurately evaluated suicide risk than did ChatGPT-3.5.

**Methods:**

ChatGPT was tasked with assessing a vignette that depicted a hypothetical patient exhibiting differing degrees of perceived burdensomeness and thwarted belongingness. The assessments generated by ChatGPT were subsequently contrasted with standard evaluations rendered by mental health professionals. Using both ChatGPT-3.5 and ChatGPT-4 (May 24, 2023), we executed 3 evaluative procedures in June and July 2023. Our intent was to scrutinize ChatGPT-4’s proficiency in assessing various facets of suicide risk in relation to the evaluative abilities of both mental health professionals and an earlier version of ChatGPT-3.5 (March 14 version).

**Results:**

During the period of June and July 2023, we found that the likelihood of suicide attempts as evaluated by ChatGPT-4 was similar to the norms of mental health professionals (n=379) under all conditions (average *Z* score of 0.01). Nonetheless, a pronounced discrepancy was observed regarding the assessments performed by ChatGPT-3.5 (May version), which markedly underestimated the potential for suicide attempts, in comparison to the assessments carried out by the mental health professionals (average *Z* score of –0.83). The empirical evidence suggests that ChatGPT-4’s evaluation of the incidence of suicidal ideation and psychache was higher than that of the mental health professionals (average *Z* score of 0.47 and 1.00, respectively). Conversely, the level of resilience as assessed by both ChatGPT-4 and ChatGPT-3.5 (both versions) was observed to be lower in comparison to the assessments offered by mental health professionals (average *Z* score of –0.89 and –0.90, respectively).

**Conclusions:**

The findings suggest that ChatGPT-4 estimates the likelihood of suicide attempts in a manner akin to evaluations provided by professionals. In terms of recognizing suicidal ideation, ChatGPT-4 appears to be more precise. However, regarding psychache, there was an observed overestimation by ChatGPT-4, indicating a need for further research. These results have implications regarding ChatGPT-4’s potential to support gatekeepers, patients, and even mental health professionals’ decision-making. Despite the clinical potential, intensive follow-up studies are necessary to establish the use of ChatGPT-4’s capabilities in clinical practice. The finding that ChatGPT-3.5 frequently underestimates suicide risk, especially in severe cases, is particularly troubling. It indicates that ChatGPT may downplay one’s actual suicide risk level.

## Introduction

### Background

Large language models (LLMs), a subset of natural language processing (NLP) models, are trained with ample textual data to generate advanced language predictions [1]. Recently, a ChatGPT-based agent from OpenAI in California gained significant internet attention due to its ability to produce human-like text from varied prompts [2,3]. In fact, since its launch in November 2022, ChatGPT swiftly gained millions of users [1]. Its ability to handle complex tasks and generate human-like language marks a breakthrough in artificial intelligence (AI) and natural language processing [3,4]. However, despite extensive study in academia, its applications in applied psychology [5-7], particularly its efficacy in addressing critical mental health issues such as suicide prevention, remain unclear.

Suicide represents a significant global health issue and a leading cause of mortality [8,9]. Despite numerous comprehensive studies over the past few decades, the issue of suicide risk assessment remains unresolved in the mental health field [10,11]. The currently used questionnaires and clinical evaluations have yet to overcome notable psychometric challenges [12]. Another difficulty is the lack of communities’ sufficient access to suicide risk assessment [13]. In an extensive review of linguistic markers linked to suicidal tendencies, 75 studies involving 279,032 individuals were analyzed. Suicidal ideation was associated with increased use of intensifiers and superlatives, while suicidal actions correlated with more pronouns, varying verb usage, and other specific linguistic patterns [14]. Fernandes et al [15] used a machine learning algorithm on electronic health records to detect “suicidal thoughts” or “suicide attempt” mentions. Although successful, the algorithm’s ability to predict future suicidal actions is yet uncertain. Aladağ et al [16] used machine learning on 10,000 forum posts classified by a clinician for suicidal content. Using metrics from the Linguistic Inquiry and Word Count software, they achieved high prediction accuracy, but did not specify the key metrics. Similarly, Tadesse et al [17] analyzed Reddit posts using NLP, comparing those with and without suicidal content without clinical validation.

A previous study evaluated the potential of ChatGPT-3.5 (March 14, 2023, version), a linguistic AI model, versus mental health professionals in assessing suicide risk [6]. The results showed that ChatGPT-3.5 (March 14 version) generally underestimated the risk of suicide, which raised concerns about its reliability for such assessments. Thus, our previous study indicated that until further evidence could support its accuracy, mental health professionals should consider ChatGPT’s assessments to be nonprofessional [6]. However, considering recent developments, including the launch of ChatGPT-4 and the release of a new version of ChatGPT-3.5 (May 24, 2023, versions of both), we aimed in the previous study to examine whether these new versions exhibited improvements over their predecessors.

In recent times, the potential of AI to augment mental health services has been the subject of intense scrutiny. The envisaged applications range from aiding diagnostics [18], to streamlining administrative tasks that would afford clinicians more patient time [19], to enhancing social motivation and attentional performance via AI-powered games that foster mental health [20]. A recent review [21] shed light on the prospective utility of AI-driven chatbots in the mental health sphere.

In terms of linguistic diversity, ChatGPT-4 exhibits enhanced multilingual abilities [22,23] compared to ChatGPT-3.5. Specifically, it represents a substantial advance in model progression, with amplified capabilities such as multilingual expertise, extended context length, and image processing, thus presenting intriguing possibilities across diverse fields [24]. Yet the cost of ChatGPT-4 and its inherent limitations underline the necessity of scrutinizing a specific application before choosing it [22]. It should be noted that ChatGPT-4 has been acknowledged for its superior performance relative to its antecedents [25]. In a study evaluating ChatGPT-4’s performance on the Ophthalmic Knowledge Assessment Program examination versus its predecessor, ChatGPT-3.5, the results demonstrated that ChatGPT-4 notably outperformed ChatGPT-3.5 (81% vs 57%), indicating progress in medical knowledge evaluation [26]. Another study contrasting the performance of ChatGPT-3.5 and ChatGPT-4 on the Japanese Medical Licensing Examination revealed that ChatGPT-4 surpassed ChatGPT-3.5 in terms of accuracy, particularly with regard to general, clinical, and clinical sentence queries [27].

### This Study

The capacity of clinicians to identify signs of potential suicide is paramount for the administration of proper crisis management and suicide intervention tactics, particularly during times of severe crisis [28]. Given the seriousness of the issue, a substantial commitment toward evaluating suicide risk is vital [29-31].

AI, in theory, could assist gatekeepers in their decision-making processes and enhance the efficacy of formal psychometric tools and clinical evaluations in predicting suicidal behavior. Current methods often fall short in their predictive capabilities [29,32,33].

In this study, we evaluated the ability of ChatGPT-3.5 and ChatGPT-4 (May 24 versions) to identify the risk of suicide based on the interpersonal theory of suicide (ITS), an established and empirically backed theoretical model proposed by Joiner et al [34,35] for assessing suicide risk and identifying associated factors. We performed these evaluations on the same day. We specifically evaluated how the 2 key dimensions of ITS—perceived burdensomeness and thwarted belongingness—influence therapists’ perceptions and assessments of suicidal ideation and behavior. Perceived burdensomeness refers to a psychological state wherein individuals feel their existence imposes a burden on their family, friends, or society. This perception leads individuals to believe their death might be more beneficial than their continued existence [34]. Thwarted belongingness, as defined by Van Orden et al [35], alludes to feelings of alienation from others and emphasizes the distressing sensation of exclusion from one’s family, friends, or other valued groups. This construct plays a critical role in suicide, as belongingness represents a fundamental psychological need, and failure to meet this need can lead to profound distress [36]. In a previous study, the tendency of ChatGPT-3.5 (March 14 version) to consistently underestimate the risk of suicide attempts, compared to estimates by professionals in the mental health field, was unveiled [6]. This trend was maintained irrespective of the influence of resilience, perceived burdensomeness, or thwarted belongingness on the evaluation of suicidal ideation and the potential risk of a suicide attempt. Alarmingly, we observed that the extent of underestimation of suicide risk by ChatGPT-3.5 (March 14 version) was at its highest when the severity of the case was at its maximum. This striking discovery poses a significant point of concern.

### The Objectives of This Study

We aimed to (1) assess the ability of ChatGPT-3.5 and ChatGPT-4 (May 24 versions) to evaluate suicidal behavior and risk factors across 2 identifiable variables—perceived burdensomeness and thwarted belongingness—compared to the ability of mental health professionals and to the earlier version of ChatGPT-3.5 (March 14 version) and (2) evaluate whether ChatGPT-4 (May 24 version) evaluates suicide risk more accurately than ChatGPT-3.5 (May 24 version).

## Methods

### AI Procedure

Using ChatGPT-4 and the free version of ChatGPT-3.5 (both May 24), we conducted 3 evaluations during the period of June and July 2023 to examine the ability of these two versions of ChatGPT to evaluate various aspects of suicide risk compared to the performance of mental health professionals and to the previous version of ChatGPT-3.5 (March 14).

### Input Source

In this investigation, we adopted the text vignette methodology as elucidated by Bresin et al [37] to examine the observer’s perspective on suicidal acts. A specific vignette from Levi-Belz and Gamliel [38] was entered into the ChatGPT interface. The chosen vignette contained general background details and 2 additional paragraphs; one paragraph portrayed either low or high perceived burdensomeness and the other either low or high thwarted belongingness, for a total of 4 possible combinations and 4 vignettes (Multimedia Appendix 1). Each of the vignettes was presented 3 times to both the ChatGPT-4 and ChatGPT-3.5 models, each time in a new tab.

### Measures

After each of the 4 vignette conditions, that is, (1) low burdensomeness and low belongingness, (2) low burdensomeness and high belongingness, (3) high burdensomeness and low belongingness, and (4) high burdensomeness and high belongingness, we submitted 6 questions to ChatGTP-4 and 3.5 (May 24 versions), taken from Levi-Belz and Gamliel [38]. Each question was categorized into one of four subscales: (1) psychache, that is, psychological pain (Q1: “What do you think is Jane’s level of distress?” and Q2: “What do you think is Jane’s level of mental pain?”), (2) suicidal ideation (Q3: “What is the likelihood Jane will have suicidal thoughts?”), (3) risk of suicide attempt (Q4: “What is the likelihood Jane will attempt suicide?” and Q5: “What is the likelihood Jane will make a serious suicide attempt?”), and (4) resilience (Q6: “How do you assess Jane’s level of resilience?”). All 6 questions used a 7-point Likert scale, estimating intensity (Q1, Q2, Q6) and likelihood (Q3-Q5), ranging from 1 (very slight) to 7 (very high). Multimedia Appendix 2 shows examples of ChatGPT answers to the questions.

### Scoring

The performance of ChatGPT-4 and ChatGPT-3.5 (May 24 versions) was scored and compared to the results of professional mental health professionals, which were previously published in accordance with Levi-Belz and Gamliel [38]. Subsequently, we compared the ChatGPT scores to the norms of 379 mental health professionals (n=80, 21% male; mean age 36, SD 8.8 years). Of these, 53 were graduate students in psychology, 266 held a master’s degree, and 60 held a PhD. Regarding their professional roles, 43 participants were supervisors, 108 were certified experts, 128 were interns, and 100 either had not begun their internship or were in professions that did not require an internship. The majority of the sample (n=318, 84%) were currently practicing, whereas the remaining participants had previously worked in the mental health field but were currently not working [38].

### Statistical Analysis

The data are presented as the mean (SD) scores of the first, second, and third evaluations of ChatGPT. Percentage and *Z* scores were used to evaluate the differences between the different ChatGPT versions’ performance and the norms of the mental health professionals reported by Levi-Belz and Gamliel [38].

## Results

### Overview

Table 1 depicts the ChatGPT-4 and 3.5 (May 24 versions) mean (SD) scores for all four conditions, that is, (1) low burdensomeness and low belongingness, (2) low burdensomeness and high belongingness, (3) high burdensomeness and low belongingness, and (4) high burdensomeness and high belongingness, for the four dependent variables: (1) psychache, (2) suicidal ideation, (3) risk of suicide attempt, and (4) resilience, compared to the norms of the health professionals reported by Levi-Belz and Gamliel [38] and the results of ChatGPT-3.5 (March 14 version) reported by Elyoseph and Levkovich [6].

**Table 1:** Descriptive statistics of the mental health professionals (reported by Levi-Belz and Gamlie [38]), ChatGTP-4 (May 24), ChatGTP-3.5 (May 24), and ChatGTP-3.5 (March 14) for the 4 dependent variables (risk for suicide attempt, suicidal ideation, psychache, and resilience) in the 4 conditions.

Dependent variables		Low perceived burdensomeness and low thwarted belongingness, mean score (SD)	Low perceived burdensomeness and high thwarted belongingness, mean score (SD)	High perceived burdensomeness and low thwarted belongingness, mean score (SD)	High perceived burdensomeness and high thwarted belongingness, mean score (SD)
**Risk for suicide attempt**					
	Mental health professionals	2.3 (1.0)	2.9 (1.3)	3.1 (1.2)	4.1 (1.2)
	ChatGPT 4 (May)	2.0 (0.6)	3.0 (1.5)	3.5 (0.6)	4.0 (0.6)
	ChatGPT 3.5 (May)	1.5 (0.8)	2.2 (0.7)	2.0 (0.6)	2.8 (0.7)
	ChatGPT 3.5 (March)	1.5 (0.0)	1.5 (0.0)	1.5 (0.0)	2.7 (0.5)
**Suicidal ideation**					
	Mental health professionals	3.6 (1.3)	4.3 (1.4)	5.0 (1.3)	5.4 (1.1)
	ChatGPT 4 (May)	4.0 (0.0)	4.6 (0.5)	6.0 (0.0)	6.0 (0.0)
	ChatGPT 3.5 (May)	3.3 (0.5)	4.7 (0.5)	4.3 (0.5)	5.7 (0.5)
	ChatGPT 3.5 (March)	4.0 (0.0)	4.0 (0.0)	4.0 (0.0)	5.3 (0.5)
**Psychache**					
	Mental health professionals	5.5 (0.8)	5.9 (0.6)	5.9 (0.7)	6.2 (0.7)
	ChatGPT 4 (May)	6.0 (0.0)	6.3 (0.5)	6.8 (0.4)	7.0 (0.0)
	ChatGPT 3.5 (May)	6.0 (0.0)	6.0 (0.0)	6.2 (0.4)	6.3 (0.5)
	ChatGPT 3.5 (March)	6.0 (0.0)	6.0 (0.0)	6.0 (0.0)	6.3 (0.2)
**Resilience**					
	Mental health professionals	5.1 (0.8)	4.5 (1.0)	4.2 (1.0)	3.4 (1.2)
	ChatGPT 4 (May)	4.7 (0.5)	4.0 (0.0)	2.7 (0.5)	2.3 (0.5)
	ChatGPT 3.5 (May)	4.7 (0.5)	4.0 (0.0)	2.6 (0.5)	2.3 (0.5)
	ChatGPT 3.5 (March)	3.7 (0.5)	3.3 (0.0)	3.0 (0.0)	3.0 (0.0)

### Risk of Suicide Attempt

**Figure 1:**
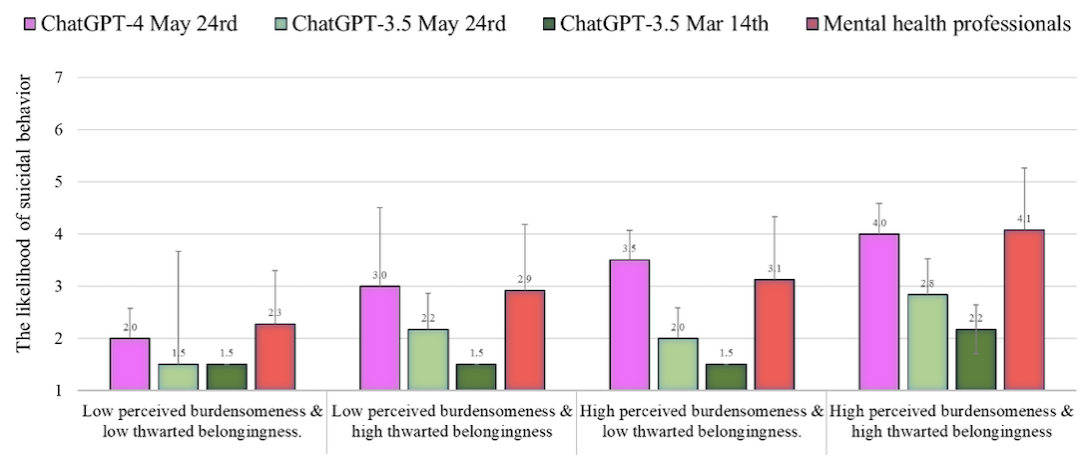
The level of risk of suicide attempts (mean, SD) assessed by ChatGPT-4 and 3.5 (May 24 versions) as compared to the norms of the health professionals and ChatGPT-3.5 (March 14 version).

Figure 1 shows the level of risk of suicide attempts as assessed by ChatGPT-4 and 3.5 (May 24 versions) as compared to the norms of the health professionals reported by Levi-Belz and Gamliel [38] and the results of ChatGPT-3.5 (March 14 version) reported by Elyoseph and Levkovich [6]. The level of risk of suicide attempts evaluated by ChatGPT-4 was similar to that of the mental health professionals in all conditions (*t*_86_=0.13-0.57, *P*=.56-.9; average *Z* score=+0.01, average *Z* score in absolute value=0.17). In contrast, ChatGPT-3.5 (May 24 version) provided a significantly lower assessment of the level of risk of suicide attempts than did the mental health professionals (percentile range 15-27; average *Z* score=–0.83; average *Z* score in absolute value=0.83). This underestimation is similar to what was found by the ChatGPT-3.5 March 14 version (percentile range 5-23; average *Z* score=–1.21; average *Z* score in absolute value=1.21), but to a lesser extent.

### Suicidal Ideation

**Figure 2:**
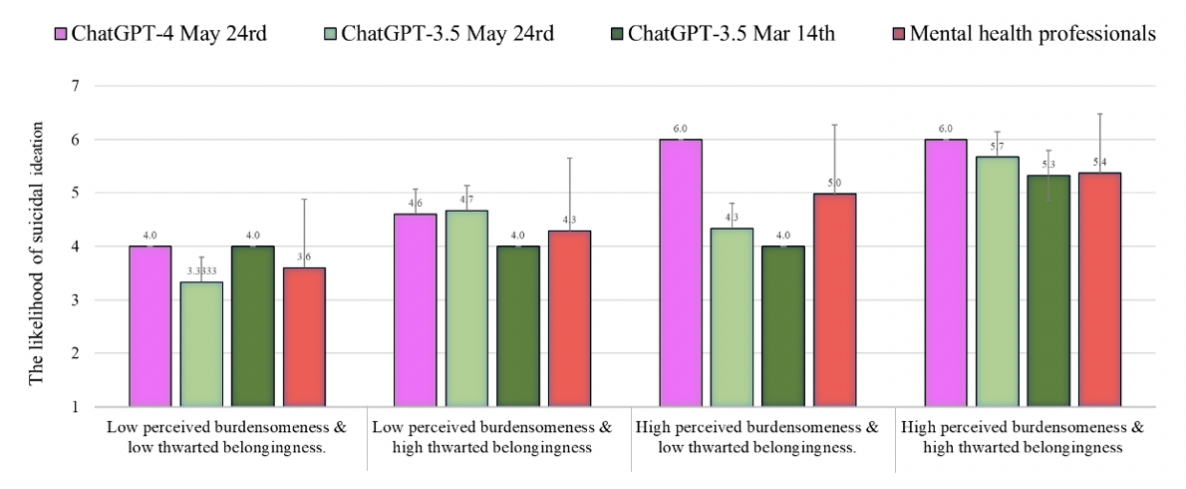
The likelihood of suicidal ideation (mean, SD) assessed by ChatGPT-4 and 3.5 (May 24 versions) as compared to the norms of the health professionals and ChatGPT-3.5 (March 14 version).

Figure 2 shows the level of risk of suicidal ideation as assessed by ChatGPT-4 and 3.5 (May 24 versions) as compared to the norms of the health professionals reported by Levi-Belz and Gamliel [38] and the results of ChatGPT-3.5 (March 14 version) reported by Elyoseph and Levkovich [6]. ChatGPT-4 evaluated the likelihood of suicidal ideation higher than the mental health professionals in all conditions (percentile range 59-78; average *Z* score=0.47, average SD in absolute value=0.47). In contrast, ChatGPT-3.5 (May 24 version) assessed the likelihood of suicidal ideation quite similarly to the way in which the mental health professionals did (percentile range 31-61; average *Z* score=–0.04; average *Z* score in absolute value=0.31). This estimation was also quite similar to the results of the ChatGPT-3.5 March 14 version (percentile range 22-62; average *Z* score=–0.17; average *Z* score in absolute value=0.33).

### Psychache

**Figure 3:**
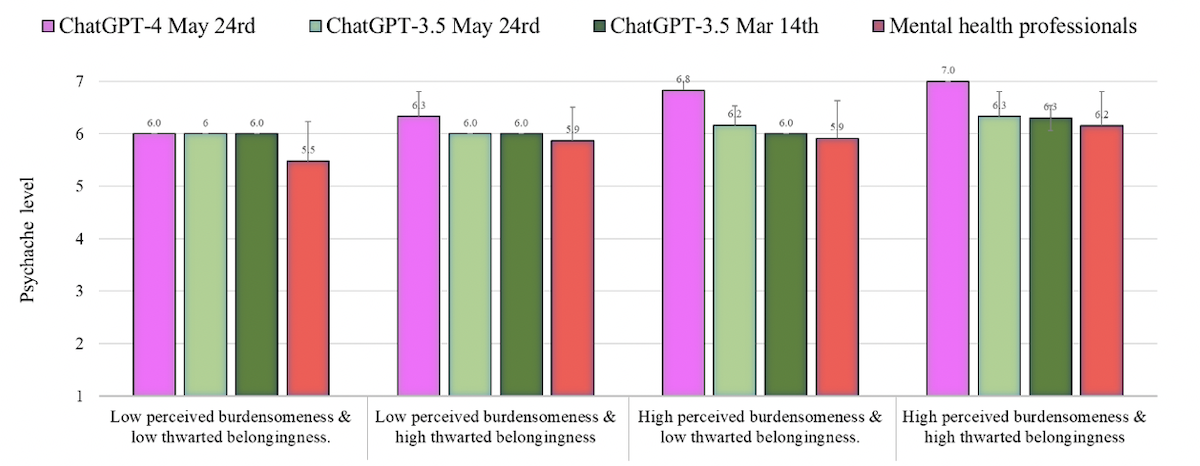
The level of psychache (mean, SD) assessed by ChatGPT-4 and 3.5 (May 24 versions) as compared to the norms of the health professionals and ChatGPT-3.5 (March 14 version).

Figure 3 shows the level of psychache as assessed by ChatGPT-4 and 3.5 (May 24 versions) as compared to the norms of the health professionals reported by Levi-Belz and Gamliel [38] and the results of ChatGPT-3.5 (March 14 version) reported by Elyoseph and Levkovich [6]. The level of psychache evaluated by ChatGPT-4 was higher than the level evaluated by the mental health professionals in all conditions (percentile range 76-90; average *Z* score=1.00, average SD in absolute value=1.00). In contrast, ChatGPT-3.5 (May 24 version) assessed the level of psychache quite similarly to how it was assessed by the mental health professionals (percentile range 55-76; average *Z* score=0.38; average *Z* score in absolute value=0.38). This estimation was also quite similar to the results of the ChatGPT-3.5 March 14 version (percentile range 22-62; average *Z* score=–0.17; average *Z* score in absolute value=0.31).

### Resilience

**Figure 4:**
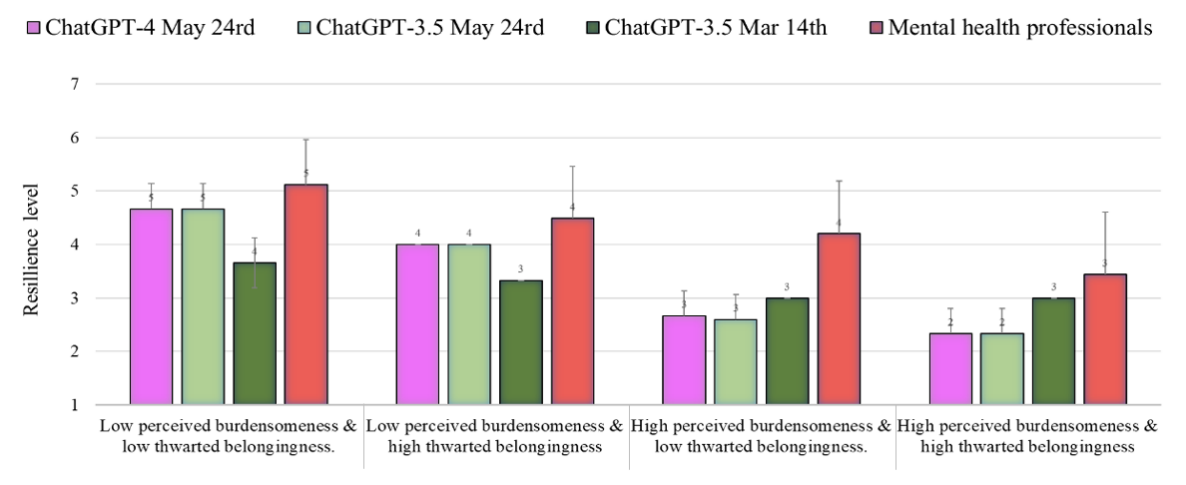
The level of resilience (mean, SD) assessed by ChatGPT-4 and 3.5 (May 24 versions) as compared to the norms of the health professionals and ChatGPT-3.5 (March 14 version).

Figure 4 shows the level of resilience as assessed by ChatGPT-4 and 3.5 (May 24 versions) as compared to the norms of the health professionals reported by Levi-Belz and Gamliel [38] and the results of ChatGPT-3.5 (March 14 version) reported by Elyoseph and Levkovich [6]. The level of resilience evaluated by ChatGPT-4 was lower than the level evaluated by the mental health professionals in all conditions (percentile range 6-31; average *Z* score=–0.89, average SD in absolute value=0.89). Similarly, ChatGPT-3.5 (May 24 version) provided a lower assessment of resilience than did the mental health professionals (percentile range 5-31; average *Z* score=–0.90; average *Z* score in absolute value=0.90). This estimation was quite similar to the results of the ChatGPT-3.5 March 14 version (percentile range 4-30; average *Z* score=–1.13; average *Z* score in absolute value=1.13).

## Discussion

### Principal Findings

In this study, we aimed to examine the efficacy of ChatGPT in conducting suicide risk assessments, with a particular focus on 2 discernible factors—sense of perceived burdensomeness and feelings of thwarted belongingness—across a period of 2 months. In addition, we wished to compare and analyze the precision of suicide risk assessment between two AI models, ChatGPT-4 and ChatGPT-3.5 (both the March 14 and May 24 versions), to ascertain which demonstrated superior performance. Parallels were found between the propensity for suicide attempts as assessed by ChatGPT-4 and as assessed by a sample of mental health professionals, under all conditions. However, there was a notable disparity between the evaluations made by ChatGPT-3.5 (both the March 14 and May 24 versions), which discernibly underestimated the risk of suicide attempts, and the assessments conducted by the mental health professionals.

This particular discovery carries substantial weight given the widespread and unrestricted use of AI chatbot technology by the general public. In a cross-sectional investigation, it was ascertained that 78.4% of the respondents exhibited a willingness to use ChatGPT for the purpose of self-diagnosis [39]. In a variety of circumstances—including mental health evaluations, therapeutic consultations, medication management, and patient education—it has been substantiated that ChatGPT effectively delivers pertinent information and support to patients [40]. A recent review scrutinized the benefits conferred by ChatGPT and analogous LLMs in enhancing medical education, refining clinical decision-making processes, and propelling superior patient outcomes [41]. The findings of this study constitute preliminary evidence that ChatGPT-4 can provide an assessment similar to that provided by professionals on critical matters such as predicting the likelihood of suicidal behavior. The implications are vast; namely, ChatGPT-4 could serve as a decision-making support tool for clinicians and possibly offer professionals a second opinion. All of these possibilities necessitate continued research and development.

That said, we found that compared to the estimations made by mental health professionals across all conditions, ChatGPT-4 overestimated suicidal ideation. Surprisingly, the evaluation of suicidal ideation by ChatGPT-3.5 (May 24 version) was similar to the assessments made by the mental health professionals in all conditions as compared to the assessments of ChatGPT-4. Notably, these assessments by the May 24 version of ChatGPT-3.5 were consistent with the findings obtained from the March 14 version of the same ChatGPT-3.5 model.

The ability to competently use assessments identifying suicidal ideation in client interactions is of utmost importance for mental health professionals. It is estimated that approximately 1 in 4 such professionals will encounter client suicide [42]. The accurate discernment of suicidal ideation thus marks a fundamental stride toward its prevention and efficient management. Although no tool can guarantee absolute certainty, having access to reliable instruments for clinical assessment can prove indispensable for practitioners. Standardized assessments ought to be used in tandem with a clinical interview, mnemonic devices, and the evaluation of risk factors in order to conduct a comprehensive appraisal of patient risk [43]. Although it is evident that ChatGPT-4 showed a tendency to overestimate suicidal ideation compared to the mental health professionals, it did so by only a small margin. Currently, we do not know whether this gap resulted from the AI’s overestimation or from mental health professionals’ underestimation.

The findings of this study highlight that there was a higher degree of psychache, as assessed by ChatGPT-4, than that rendered by a group of mental health professionals in all observed conditions. By contrast, the assessment of psychache by the May 24 version of ChatGPT-3.5 aligned closely with the mental health professionals’ assessment. The ChatGPT-3.5 (May 24 version) findings are congruent with the outcomes from its March version. A potential explanation for these findings might be that ChatGPT-4 tends to overestimate certain metrics. Furthermore, these findings suggest that different ChatGPT versions should still be used in a balanced manner, not exclusively, but in combination with the professional expertise of health care practitioners. This suggestion seems to align with the positions of professionals regarding the use of ChatGPT. In a survey assessing health care workers’ interactions with ChatGPT, a significant majority of respondents (75.1%) expressed comfort with the idea of integrating ChatGPT into their health care practice, including in the aiding of medical decision-making (39.5%) [44].

Contrary to the other findings, this study indicates that the level of resilience as evaluated by ChatGPT-4 was lower than the level as assessed by the mental health professionals in all conditions. Similarly, ChatGPT-3.5 (March and May) provided a lower assessment of the level of resilience than did the mental health professionals. Despite the common use of the resilience concept, it should be noted that it has different definitions and a variety of measurement methods [45]. Resilience is a multifaceted concept shaped by various elements that span the individual, environmental, organizational, and cultural spheres [46]. This inherent complexity and multidimensionality renders resilience a challenging construct to operationalize and measure [46]. Experienced professionals, when confronted with case descriptions, are likely to consider an amalgamation of these factors, exhibiting a nuanced understanding that may be challenging to encapsulate fully in technological iterations. Thus, although the different versions of the technology offer significant value, their assessments must be contextualized within this wider understanding of resilience, highlighting the importance of a balanced approach that includes professional insight.

This research highlights the complexity of evaluating an individual’s risk for suicide. The foregoing evidence demonstrates the potential advantages of using ChatGPT-4 to bolster clinical decision-making in the realm of suicide risk assessments among professionals [18,19,21]. Furthermore, ChatGPT-4 could play a crucial role in enhancing training and clinical procedures among mental health and medical professionals [26,27,47]. The easy access to ChatGPT and the possibility of reducing feelings of stigma may in the future drive the use of mental health assessment services by the general public.

However, the incorporation of ChatGPT into suicide risk detection mechanisms also presents a series of complexities. The reliability of ChatGPT predictions is intrinsically linked to the quality and demographic inclusivity of the training data [31]. Data biases or inadequate demographic representation could lead to erroneous predictions or exacerbate existing health disparities. Moreover, ChatGPT algorithms often function as opaque entities, obscuring the reasoning behind their predictive mechanisms. This lack of clarity can impede the development of trust and acceptance among users [48]. The deployment of ChatGPT for the identification of depression raises several ethical issues [48]. Ensuring data privacy and security is paramount, particularly given the sensitive nature of mental health information [49]. Research on the ethical facets of mental health within the broader populace suggests that participants express reservations about the widespread acceptance of AI and the implications of its capabilities for human welfare. Furthermore, there is apprehension regarding the potential for medical inaccuracies [50]. Moreover, there is expressed concern from patients regarding the potential discriminatory implications stemming from the use of AI [51]. The potential misuse that could exacerbate health disparities is an imperative issue. There is also the necessity of respecting patient autonomy, as AI can spread misleading medical information or endorse unverified treatments, which may compromise a general practitioner’s understanding of medical conditions [52]. While AI offers the unique capability to craft authentic patient scenarios—thus enhancing medical training—it is vital to remain cognizant of the risks associated with the misuse or misrepresentation of these tools [52]. Users must be thoroughly informed about the use and protection of their data. Importantly, ChatGPT should not replace human clinical judgment in the diagnostic process, but rather supplement it, thereby aiding professionals in making more-informed clinical decisions.

### Limitations

This study, although informative, is not without limitations. To begin with, the assessment of the risk of suicide is based on a limited number of vignettes that centered on a woman in relatively stable condition of an age bracket not typically associated with high suicide risk and with no history of previous suicide attempts. Such a narrow representation does not adequately encapsulate the broad spectrum of suicide risk. As a result, we suggest that future research should expand to include a more diverse demographic. Vignettes should be created featuring varied demographics, such as male participants, individuals with psychiatric conditions, adolescents, and the elderly, to ensure a more holistic understanding of suicide risk. Secondly, our comparison of ChatGPT data was limited to a sample drawn from mental health professionals in Israel. To achieve a more comprehensive evaluation and to understand cross-cultural nuances in suicide risk assessment, we advocate for the exploration of therapists’ assessments from various global contexts. Additionally, we used norms from a study about professionals as published by Levi-Belz and Gamliel [38]. We were unable to compare other statistical characteristics between the groups. We recommend further examination between an existing sample and ChatGPT. A further limitation of our study was that it relied heavily on the ITS as a theoretical basis. Although this theory provided a useful framework, it could potentially have constrained the breadth of our understanding. Moreover, our research delved into the complex domain of suicide risk assessment via AI; however, to gain more extensive insights, additional studies are required. Such studies should examine additional risk factors, integrate more expansive language models, assess data at different time intervals, and compare results with a broader array of clinical samples. Finally, given the rapid advances in the AI field, there is an inherent challenge in generalizing the results for stable, long-term abilities. Therefore, long-term studies are a necessity to keep pace with the evolving landscape and ensure a lasting understanding of suicide risk assessment.

### Conclusion

In summary, this study yields critical knowledge on the potential of AI chatbots, particularly ChatGPT-4, to carry out suicide risk evaluations, while also highlighting the intricacies and subtleties entailed. Even though ChatGPT-4 overassessed suicidal thoughts, it showed an assessment accuracy on par with mental health experts in regard to suicidal behaviors. This research underlines the significance of AI tools such as ChatGPT serving as a supplement rather than as a substitute for professionals. The research also illustrates the rapid development of AI in the field of applied psychology and the need for research at multiple points in time and in regard to multiple versions in order to achieve reliable results.

## Appendix

Multimedia Appendix 1 Vignette presented to ChatGPT-3.5 and ChatGPT-4.

Multimedia Appendix 2 Examples of questions and ChatGPT answers.

## Abbreviations

AI: artificial intelligence
ITS: interpersonal theory of suicide
LLM: large language model
NLP: natural language processing

## Data Availability

The data that support the findings of this study are available from the authors upon reasonable request.
