# Supplementary material for: Suicide Risk Assessments Through the Eyes of ChatGPT-3.5 Versus ChatGPT-4: Vignette Study
Source: JMIR Ment Health. 2023 Sep 20;10:e51232. doi: 10.2196/51232 (PMC10551796; doi:10.2196/51232)
Supplement: Multimedia Appendix 1 [file mental_v10i1e51232_app1.docx]

### Appendix 1: vignette was present to ChatGPT-3.5 and ChatGPT-4

### General background

*Jane is a 37-year-old single woman without children, currently living alone, but in her past spent a long time living with her father, who passed away two years previously following a severe illness. Jane holds a B.A. degree and has kept a steady job for10 years. Recently, however, her manager informed her that, due to financial cuts, she would have to dismiss her. Jane had a close, intimate relationship for the past year with a boyfriend, her first serious and meaningful one; she was very excited about the relationship, hoping it would lead to marriage. However, following a period of repeated quarrels, her boyfriend informed her he no longer loved her, and he left her. Jane was left feeling very sad and hurt. In recent weeks she keeps thinking about the relationship over and over again, trying to analyze what happened. Jane is experiencing a heavy emotional load; she is very tired, lacks energy, and finds it hard to sleep at night. Her routine tasks have become difficult for her, and she finds it hard to concentrate and to function properly. She dreads the future, and it seems to her there is no chance she’ll find a love like that again.*

### Low perceived burdensomeness

*In general, Jane feels she has abilities. She feels she has something to contribute to the world and to others around her, and sometimes people come to her for help and advice. She feels important and meaningful, and she seems to believe in herself.*

### High perceived burdensomeness

*Jane feels worthless. Often she experiences herself as a burden to the people around her and feels that it would be better if she were gone. She often sees herself as having no real meaning in other people’s lives and she experiences feelings of self-hatred. For example, she is angry at herself and thinks that the departure of her boyfriend was her fault.*

**Low thwarted belongingness**

*Jane has some close friends, and she feels that she can tell them about what she has been going through in recent weeks. In general, she makes connections relatively easily and, despite the recent crisis, she feels lucky because there are people around who care for her.*

**High thwarted belongingness**

*Jane is experiencing significant loneliness in general and she has few, if any, close friends around her. She is very withdrawn, distant from others, and feels that there are no people to whom she can turn in times of trouble. Therefore, she has not shared her feelings with anyone since the breakup.*
