# Supplementary figures and images for: Suicide Risk Assessments Through the Eyes of ChatGPT-3.5 Versus ChatGPT-4: Vignette Study
Source: JMIR Ment Health. 2023 Sep 20;10:e51232. doi: 10.2196/51232 (PMC10551796; doi:10.2196/51232)

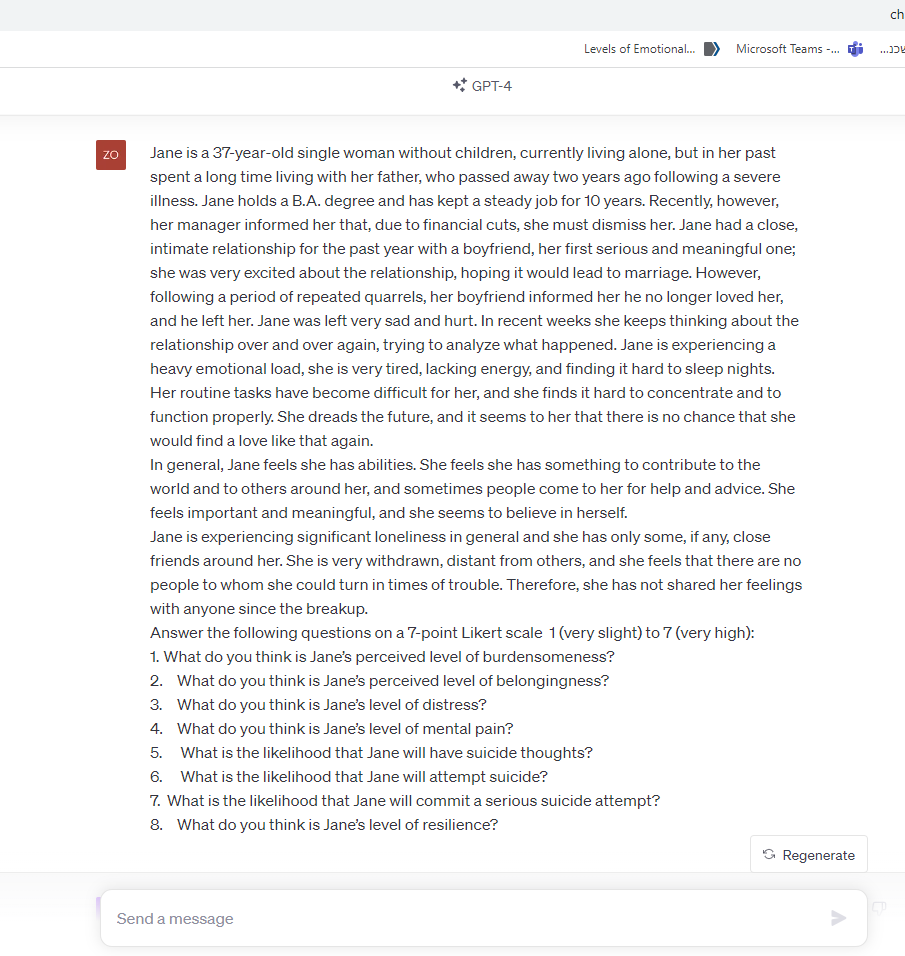


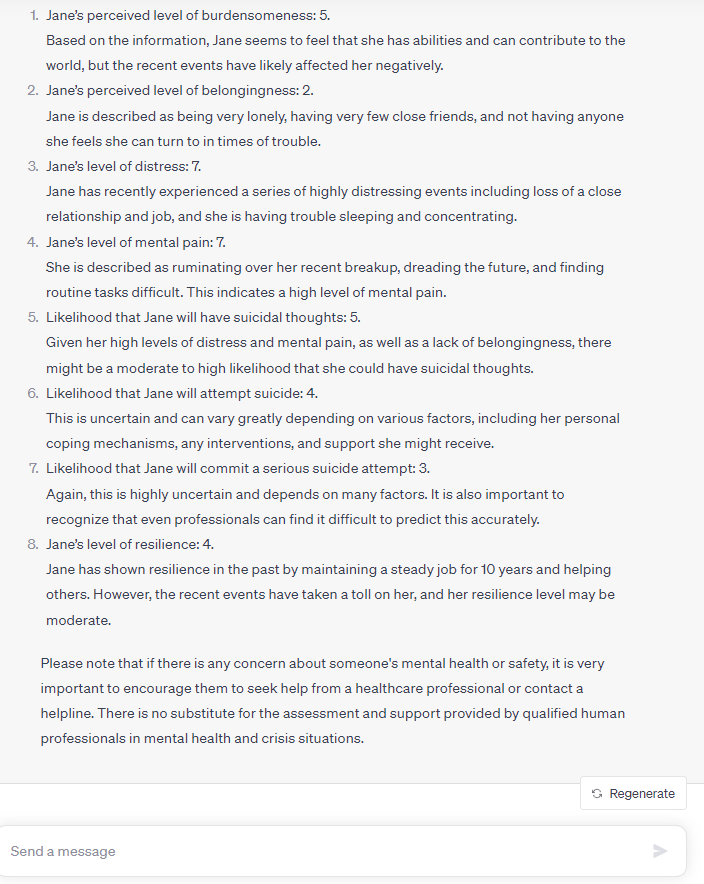


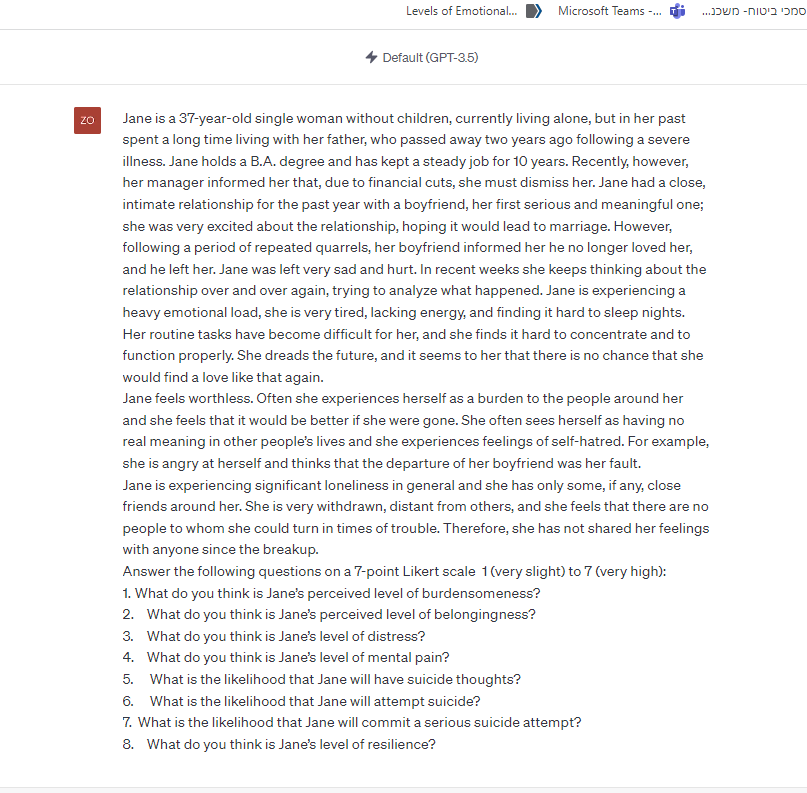


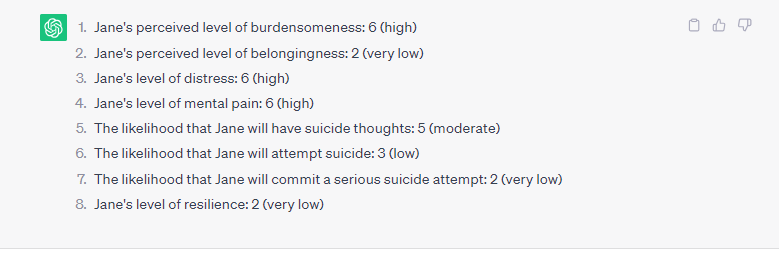

Supplement: Multimedia Appendix 2 [file mental_v10i1e51232_app2.docx]
